# Conceptualizing emergent animal farming and infectious diseases: a One Health framework

**DOI:** 10.1093/emph/eoaf029

**Published:** 2025-10-14

**Authors:** Robin Bendrey, Doaa Elkholly, Guillaume Fournié

**Affiliations:** School of History, Classics and Archaeology, University of Edinburgh, Edinburgh, UK; School of History, Classics and Archaeology, University of Edinburgh, Edinburgh, UK; Veterinary Epidemiology, Economics and Public Health Group, Department of Pathobiology and Population Sciences, Royal Veterinary College, University of London, Hatfield, UK; Veterinary Epidemiology, Economics and Public Health Group, Department of Pathobiology and Population Sciences, Royal Veterinary College, University of London, Hatfield, UK; Université de Lyon, INRAE, VetAgro Sup, UMR EPIA, Marcy l’Etoile, France; Université Clermont Auvergne, INRAE, VetAgro Sup, UMR EPIA, Saint Genes Champanelle, France

**Keywords:** One Health, infectious diseases, domestication, multispecies ecology, wildlife farming, public health

## Abstract

**Background and Objectives:**

The origin of animal farming is associated with major and inter-related changes in the ecology of humans and animals and new opportunities for pathogens to invade and be sustained in both populations. Understanding these transitions is critical for unravelling the origins and evolution of infectious diseases linked to emergent farming. This study aims to leverage One Health approaches, which recognize the inter-dependencies between the health of humans, animals, and environments, to better understand the ecology of humans, animals, and pathogens during the onset of farming.

**Methodology:**

This study develops a One Health conceptual framework to explore the interconnected ecological and health impacts of early animal farming. It employs archaeological and contemporary wildlife farming case studies to build this framework.

**Results:**

One Health frameworks are ideal to situate these changing human-animal-environment relationships in their widest context, allowing interacting processes and their feedback loops to be considered in integrated ways. Combined evaluation of ancient and contemporary emergent farming contexts enables a more inclusive approach, allowing a broader range of ecological and evolutionary insights to be considered.

**Conclusions and Implications:**

One Health approaches offer a valuable framework for understanding the historical emergence and impact of infectious diseases within farming contexts. By situating ancient interspecies relationships within broader ecological and health contexts, this framework helps investigate complex archaeological contexts and offers useful parallels to contemporary issues in wildlife farming. Insights gained from studying ancient farming systems can inform current health and agricultural policies and contribute to preventing future infectious disease outbreaks.

## INTRODUCTION

From the early Holocene, the emergence of animal farming brought with it major and inter-related shifts in the ecology of interacting humans, animals and microbial taxa. The origins of agriculture was a fundamental transition in food production strategies, and the subsequent food surpluses it could produce, and larger populations it could support, in time enabled the emergence of the contemporary human world [1–3]. Farming brought humans and animals into closer, more intense and more entangled relationships, creating conditions promoting the transmission of pathogens across and their adaptation to novel host species, and their permanent maintenance in these larger populations of interacting humans and animals [2, 4–6].

The emergence of animal farming and animal domestication are strongly related but different and offset processes. Farming, often termed agriculture, is the practice of food production through cultivating plants and raising animals [1]. Whilst farming primarily involves domesticates, it does not exclusively so [7], and as an emergent process can impose the selective pressures on wild animal populations that create the evolutionary processes humans identify as ‘domestication’ [8]. Domestication involves changes in the genotype and phenotype of the animals [9–12], some of which will also have impacts on pathogen transmission and evolutionary dynamics.

There has been considerable academic focus on the origins of animal domestication and agriculture on one hand, and past infectious diseases (particularly zoonoses) on the other. Analyses of infectious diseases are often focussed on the reconstruction of long-term infectious agent phylogeny based on available genetic sequences [e.g. 6, 13], rather than looking at the contextual details of host interactions. Considerations of socio-ecological interactions that shaped pathogen transmission in these past contexts have not kept pace with biomolecular developments. The socio-ecological relationships and their epidemiological outcomes, particularly in the early phases of farming, are often weakly conceptualized.

Conceptual frameworks are important for defining research strategy and focusing investigations of researchers working across multiple disciplines [e.g. 7, 14–16]. Here we explore how One Health approaches, which allow interacting processes within and across human populations, animals and their shared environment, including feedback loops, to be accounted for, can help focus the study of infectious diseases in emergent farming systems. We consider the conceptualization of the evolving interspecific relationships that created the conditions in which pathogens could expand their host range and become endemic in interacting host populations.

**Figure 1:**
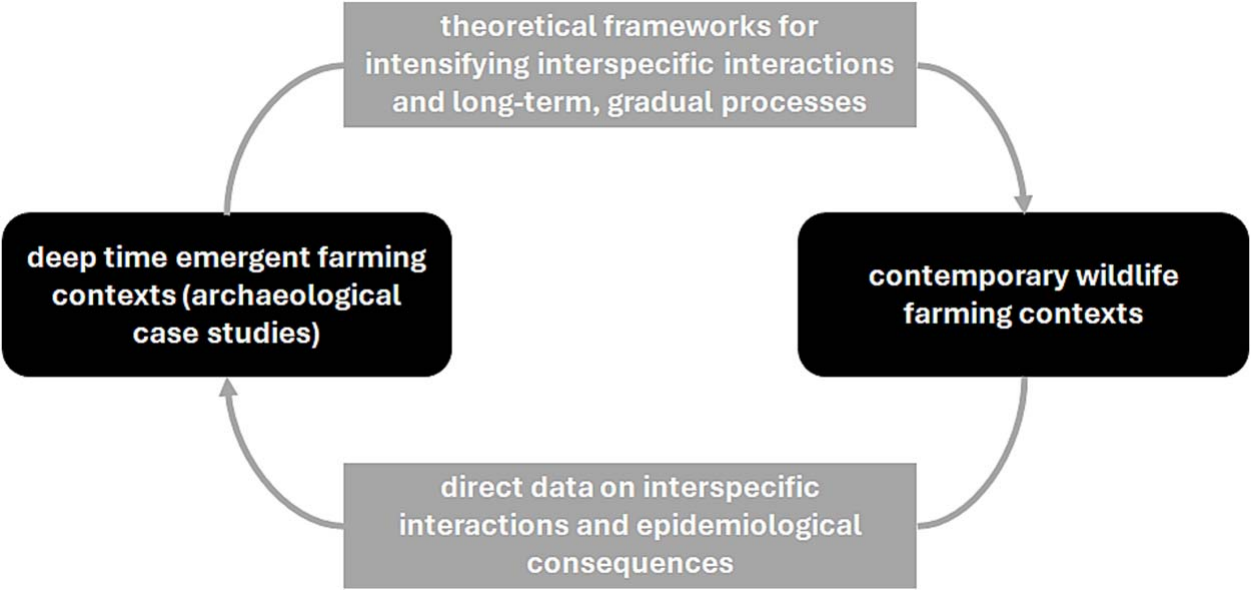
Understanding human-animal relationships and infectious diseases in emergent farming contexts—connecting past and present contexts can provide insights on health risks in emerging farming contexts. Archaeological studies of temporally-distant contexts focus on conceptualizing and evaluating evidence for subtle changes in long-term, gradual transitions, although socio-ecological dynamics and epidemiological impacts can be hard to define. In contrast, in contemporary wildlife farming (a common economic practice today involving the captive breeding of wild animals) there are direct observations of interspecific interactions and data on epidemiological consequences. This study seeks to unite insights from both within a One Health framework.

There are a number of challenges to understanding the evolution of human-animal-pathogen interactions in both the past and the present that can benefit from a long view One Health approach [17–20], an approach that has also been framed as ‘One Palaeopathology’ [21]. Archaeological domestication studies of temporally-distant contexts tend to be strongly theorized and focussed on conceptualizing and evaluating evidence for subtle changes in long-term, gradual transitions; however, epidemiological impacts are less clear due to incomplete, ‘fuzzy’ data. To contribute to the evaluation presented here, we will explore whether contemporary case studies on wild animals brought into farming contexts can provide comparators for sharpening focus on past processes (Fig. 1).

Today, ‘wildlife farming’, the captive breeding of wild animals, is a common economic undertaking, encompassing a diversity of practices and infectious disease risks [22, 23]. Whilst the place of ‘farmed’ wild animal populations in the modern world is often understudied and consequently less well understood and valued, some case studies have provided detailed data and understanding on interspecific interactions and epidemiological consequences. Following Altman and Mesoudi [24], the aim here of exploring contemporary contexts is: 1) to demonstrate that the conceptual framework discussed for the past is relevant for contemporary issues and events, and 2) to allow us to make novel arguments or insights not solely apparent from examining past contexts alone (Fig. 1). It is acknowledged that the scale and intensity of contemporary and archaeological emergent farming systems will vary, and that emerging activities may be relatively informal and unregulated.

This paper evaluates how One Health approaches can contribute to the investigation of infectious disease dynamics in emergent farming contexts. Whilst the focus is on improving understanding of these processes in archaeological context, we will evaluate the potential for ancient and modern systems to inform each other (Fig. 1), through a consideration of selected wildlife farming case studies.

## ARCHAEOLOGICAL EMERGENT FARMING, ANIMAL DOMESTICATION, AND ONE HEALTH

**Figure 2:**
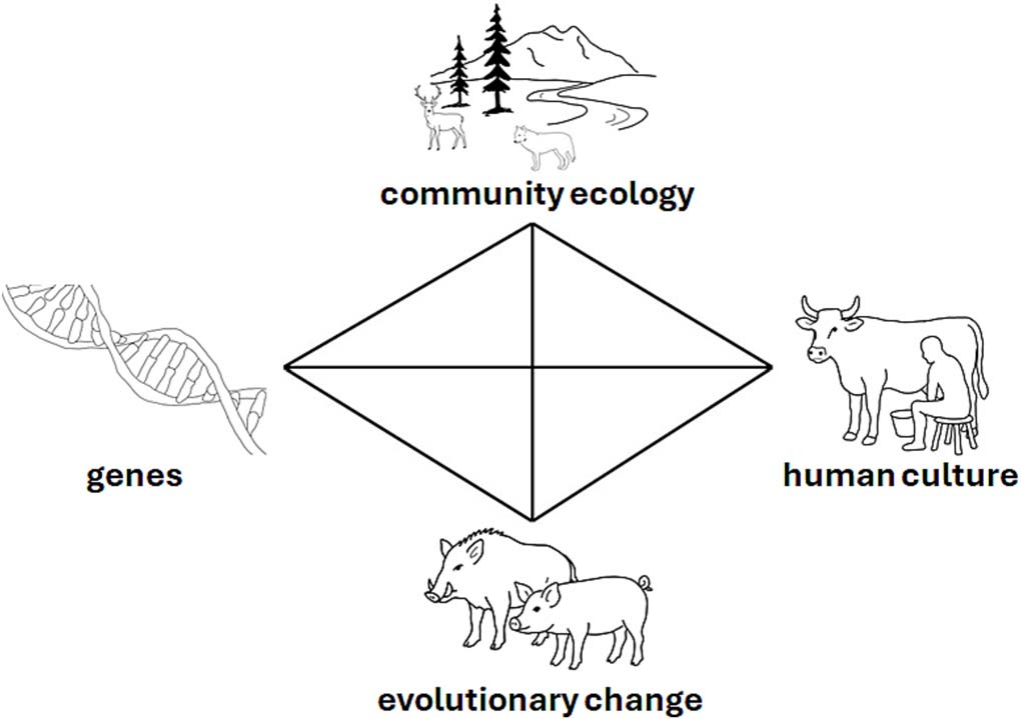
Intersections of key research areas associated with infectious diseases in emergent multispecies farming. Emergent farming may be viewed as a gene-human culture co-evolutionary process, where genetic change in the managed species and culturally transmitted human knowledge and behaviours (here illustrated by dairying practices) may both shape each other. Community ecology (the interactions of different species within a specific environment) creates the conditions promoting the transmission of pathogens and their adaptation to novel host species, and in an emergent farming context can bring about evolutionary changes in both the managed animal populations and parasitic microbial taxa impacting genotype and phenotype.

The transition from living as hunter-gatherers to farmers represents a major shift from food procurement to food production [1, 7]. With this came significant reorientation and restructuring of relationships with plants and animals involving the emergence of domestic animals, characterized by changes in their genotypes, phenotypes, ecology, and population dynamics compared to their wild counterparts. Debate surrounds the causal factors for the emergence of animal farming [1, 11], however most models posit the earliest animal domestications emerging gradually and, at least initially, unconsciously via niche-constructing behaviours by both humans and animals alike [8, 12, 25]. Domestication has also been framed as a co-evolutionary process—on the one hand we witness evolutionary change in animal populations and on the other cultural evolution of human communities. As such, agriculture may be viewed as an example of gene-culture co-evolution where the genetic evolution of domestic species and culturally transmitted human practices are both shaping each other [24] (Fig. 2).

Compared to the earlier domestication processes, the later ones are often viewed as the speedier outcome of deliberate human intent, in that once domestic animals had emerged in one form, humans could envisage other wild animals as desirable domesticates [12]. These different pathways may be illustrated by two strongly contrasting examples: 1) dogs were the first domestic animal and are thought to have in part emerged out of commensal behaviours of grey wolf (*Canis lupus*) populations; 2) all domestic golden hamsters (*Mesocricetus auratus*) derive from a single pair of siblings captured from the Syrian Desert in 1930 [25, 26]. Research on emergent models of domestication tends to study the process through focussing on changes clarified into pair-wise relationships—i.e. the interaction of two species (domesticator and domesticate) and how their relationship changed through time [10], excluding wider ecosystem considerations. This approach enables the focussed study of the emergence of a given domestic animal but neglects the study of the wider ecosystem, the outcomes of ecological intersections, and how the ecosystems make these domestication processes possible, or not, in the first place and influence their trajectory.

**Figure 3:**
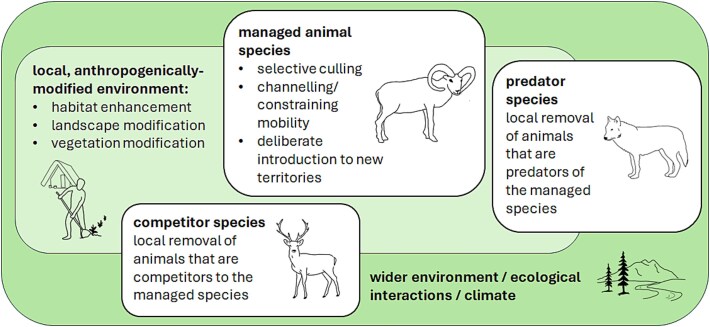
Examples of human niche constructing hunter-gatherer animal management strategies from which early animal farming may have emerged, including factors that promote the presence and sustainability of a specific taxon [27–29], and which we can potentially follow through into determinants of disease risk in early agricultural ecosystems (see Fig. 4).

As farming emerged gradually out of existing ecological relationships with plants and animals [7, 8, 12], we can consider how it disrupted these ecological relationships. A range of niche constructing activities are reported for hunter-gatherer communities, some of which contributed to the emergence of domesticates and farming systems [11, 27, 28]. Figure 3 summarizes selected management strategies from which early animal farming may have emerged, and which we can potentially follow through into determinants of disease risk in agricultural ecosystems. Examples of niche enhancement are known from hunter-gatherers introducing animal populations to islands that can be hunted [28].

Such changing ecological interactions may have shaped diverse evolutionary outcomes [30, 31], some of which we separate out as domestication [8] and others that will have impacted pathogen evolution [32] (Fig. 2). As noted above, significant advances have been made in our understanding of infectious disease outcomes associated with early animal farming through the recovery and analysis of ancient pathogen DNA [e.g. 33–34]. However, understanding ecological interactions has not kept pace with these developments. Wolfe *et al*.’s [35] conceptual model of the origins of major human infectious diseases, for example, prioritizes the evolutionary stages through which a pathogen originally exclusively infecting animals may have become transformed into one exclusively infecting humans, rather than the socio-ecological contexts of these transformations, or the possibility of reverse zoonoses. Taking an explicitly multispecies lens would enable the consideration of a wider community ecology context containing multiple actors [36, 37], which is approached here though a One Health framework [17, 20].

One Health focuses on improving health understanding and outcomes through interdisciplinary and multisectoral approaches [38, 39]. They are ideal to help situate domestication studies in their widest context to understand how socio-ecological changes influence health outcomes, conceptually moving beyond a narrow anthropocentric health focus [16]. Further, One Health frameworks of contemporary health scenarios often contain limited or no time depth, which can add valuable context. Recent work has begun to add significantly to this space by focusing on One Health in long-term perspective [e.g. 18–21]. This expands the typical temporally-shallow focus of One Health approaches, with integrated and contextualized biological and cultural long-term records helping to provide holistic understanding of developments from natural history origins to later anthropogenic influences [17].

Farming originated in complex and dynamic multispecies socio-ecological landscapes, and analytical frameworks should fully value multispecies perspectives, in this sense being ‘equity informed’ as advocated by Stephen *et al*. [16]. One Health conceptualizations need to be able to consider pathogen flow between multiple taxa [5], and in dynamically changing domestication scenarios not be constrained by wild:domestic classificatory dichotomies. Classificatory systems that rely on wild-domestic dichotomy do not allow full evaluation of domestication scenarios [7, 40]. Conceptualization models need to be able to evaluate changes through time, as well as context dependent scenarios [16], and also be mappable against the range of recoverable datasets from past contexts [18, 41].

## CONTEMPORARY WILDLIFE FARMING CONTEXTS

There is a diversity of practices in a range of contexts that fall under the term ‘wildlife farming’ [e.g. 22, 42–43]. There is also at times some ambiguity or contradiction across disciplinary literatures regarding whether a population is considered wild or domestic, seemingly stemming from various factors including the context, origin, and characteristics of the species being farmed, and also the relatively recent history of the human management of these taxa. Here we briefly consider selected examples from the literature that illustrate different socio-ecological features of contemporary emergent farming systems.

The characteristics of a given animal population, as well as human management decisions around those animals, play important roles shaping disease dynamics. In captivity, high animal density increases contacts and transmission opportunities between individuals, and this may create conditions for the spread, and maintenance of pathogens that might not have otherwise persisted. In addition, high density can expose animals to stress, immunosuppression and consequently increase animal susceptibility to infection from circulating pathogens within these farmed populations [23, 44, 45]. This may be seen in the maintenance of rabies within populations of the greater kudu (*Tragelaphus strepsiceros*) in Namibia managed for trophy hunting and venison production [46, 47] and the circulation of avian influenza virus (AIV) in South African farmed ostriches (*Struthio camelus*) [48]. The directionality of rabies transmission pathways in Namibia is revealed by historical epidemiological data, with endemicity first established in jackal, followed by transmission to and then the maintenance of the virus in greater kudu [47].

Socioeconomic drivers of consumer and producer choices can impact the selection of species raised in fur farms, animal demography, and phenotype. Yet, these choices may affect the prevalence of zoonotic pathogens, such as *Toxoplasma gondii*, the parasitic protozoan that causes toxoplasmosis. For example, this prevalence was found to vary between different canid species on a single farm in Poland, possibly due to interspecific differences in host immune response and susceptibility to the parasite [49]. The demographic structure of farmed populations may further influence pathogen circulation, with the risk of infection by *T. gondii* found to be higher in younger animals in another study on farmed raccoon dogs (*Nyctereutes procyonoides*) in two provinces in China [50].

As part of phenotype, animal behaviour may also play a role; one which may change through time in dynamically evolving human-animal relationships. A further observation from the Polish fur farm case study [49] is potentially interesting from this perspective, in that differences in prevalence of *T. gondii* infections were noted in red fox females with different behaviour types. None of the foxes classed as fearful and indifferent were infected, compared to low levels of infection in curious or aggressive animals. Although Górecki *et al*. [49] report that differences between groups of different behaviour were statistically not significant, the observation is relevant for thinking about changes in animal behaviour through time in an emerging domestication process.

The influence of human decisions on the management of farmed animal populations and their associated environment constraints or habitat enhancements is further illustrated by the farming of rusa deer (*Cervus timorensis russa*), a significant element of Mauritius’ food production [45]. They represent the largest population of large mammals on the island, raised in both semi-free-ranging herds and intensive farms. Leptospirosis is one example of the diseases investigated by Jori *et al*. [45] in these animals, with significant associations found with animal demography (age), animal density, or location of the estates (especially those exposed to higher rainfall and temperature).

These decisions around the selection, management and location of taxa in wildlife farms may then shape exposures to other animals and the parasites they carry, with the interfaces between species being key dimensions of interspecies—and especially zoonotic—transmission events. For example, in 2002 severe acute respiratory syndrome coronavirus (SARS-CoV) appeared in Foshan, Guangdong province, China, and spread to 29 countries. Subsequent investigations indicate that it was a population of farmed masked palm civets (*Paguma larvata*) that acted as the intermediate animal host transmitting the virus from its reservoir in horseshoe bats (*Rhinolophus*) to humans [51, 52]. As another example, in the Polish fur farming case study mentioned above, rodents and cats were noted as having access to the farm [49], with felids notably being the definitive hosts of *T. gondii*. Animals managed extensively may be at risk of pathogen transmission from contact with a range of free-living taxa. The extensive nature of ostrich farming exposes them to risks of avian influenza virus (AIV) [48]. Indeed, avian influenza virus molecular epidemiology has shown that above mentioned outbreaks in farmed ostriches could be linked to viruses circulating in wild bird taxa in the region [48].

The wider context of human activity and how it is organized across farms also needs to be considered. Following the Covid-19 pandemic that began in late 2019, humans are now the dominant severe acute respiratory syndrome coronavirus 2 (SARS-CoV-2) host species, posing a risk to other animal species [51]. The circulation of SARS-CoV-2 in farmed mink (*Neovison vison*) illustrates a wider socio-ecological context, as well as potential for reverse zoonotic transfer and new variant emergence in contiguous populations of humans and animals [53–55]. Investigations of farmed mink management, including farm biosecurity, during the 2020 SARS-CoV-2 outbreaks that affected production in Denmark and the Netherlands, identified that shared personnel between farms and geographical clustering increased the risk of infection in mink farms [53, 54, 56].

The translocation of farmed wildlife from one region to another may also be implicated in the further spread of pathogens. This can be seen in the spread of chronic wasting disease (CWD), a fatal prion disease of wild cervids and those farmed for meat production [57]. Although the disease has been known for over 50 years in cervid populations in North America, its distribution is expanding, with long-distance movements of farmed cervids shown to facilitate its spread [58]. An example of inadvertent introduction of a disease across the Atlantic may be seen in the impact of the introduction of the grey squirrel (*Sciurus carolinensis*) into Britain on the native red squirrel (*S. vulgaris*). Although not farmed in the strict sense, grey squirrels were introduced to estates and gardens by wealthy landowners and collectors in the late 19^th^ and early 20^th^ century for aesthetic reasons [59]. Their introduction brought squirrel pox virus (SQPV), which is highly virulent to red squirrels and significantly contributed to their decline [60]. Whilst translocated populations can introduce pathogens to new populations or taxa, they might also be vulnerable to common pathogens or parasites endemic to the area to which they have been moved. For example, in North America, meningeal worm (*Parelaphostrongylus tenuis*) is a common nonpathogenic parasite of white-tailed deer (*Odocoileus virginianus*), but causes significant morbidity and mortality in aberrant hosts, such as mule deer (*Odocoileus hemionus*), introduced into endemic areas or managed alongside white-tailed deer [61, 62].

Cultural attitudes to specific animal populations shape their treatment and management, visible in contemporary (often country-based) policy-frameworks [63] and socio-economic contexts [64]. Fearnley [64], for example, highlights the blurred distinction between domestic and wild species in duck/geese flocks in China, and through the example of breeders’ and scientists’ engagements with swan geese (*Anser cygnoides*) highlights the need to rethink the utility of wild:domestic dichotomies.

## DISCUSSION

Contemporary wildlife farming scenarios illustrate some key socio-ecological, demographic and behavioural features of intensifying human-animal relationships that may contribute to infectious disease risks in different, intersecting, ways. Emergent farming practices can structure multiple animals in novel combinations and close proximity, such as co-housing of animals, leading to heightened opportunities for interspecific pathogen transmission [42, 51]. Such transmission may occur between contiguous animal populations, and also to and from humans, with the enhanced connections between human and animal populations [53–55]. In an evolutionary perspective, farming contexts represent new environmental conditions for wild animal populations and disruption to their natural ecologies. Conditions of management, immune status and stress that the animals can be under in these novel environmental contexts can increase risks of infection [44, 48]. Animals may also be kept in larger populations and novel population structures, features that may also impact infectious disease risk.

**Figure 4:**
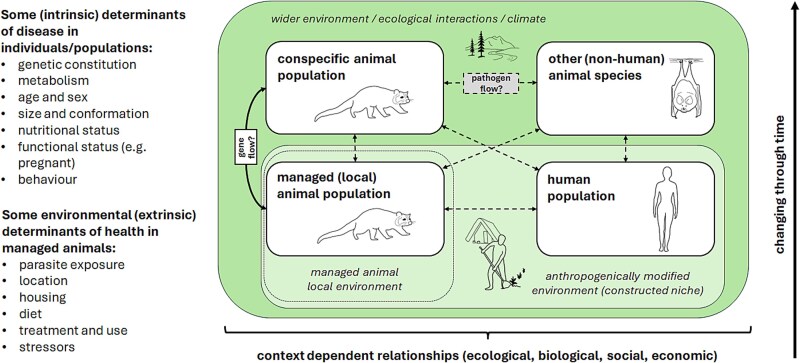
One Health conceptual framework for the study of past infectious diseases in emergent farming contexts, including selected factors (determinants) affecting the risk of infection. This seeks to enable evaluation of changing contexts for managed taxa, their connection and potential gene-flow with conspecific populations, human niche constructing behaviours, and potential pathogen flow between interacting taxa. The model allows scenarios of pathogen transmission from any direction to be considered, which as outlined in the text varies across known case studies. The illustrated taxa in Fig. 4 include farmed masked palm civets which, as the intermediate host, transmitted severe acute respiratory syndrome coronavirus (SARS-CoV) from horseshoe bats to humans in 2002 (see text).

Archaeological research focusses on the related but offset processes of farming origins and animal domestication, and engages with questions of ecological interactions and evolutionary change (Fig. 2). One Health approaches are well placed for understanding these complex, dynamic, and multispecies processes. The framework presented in Fig. 4 attempts to draw from, and be relevant for, the ancient and contemporary contexts discussed above.

Figure 4 presents a One Health conceptual framework for the study of past infectious diseases in emergent farming contexts, including gradual domestication of one, or more, animal populations and their connection and potential gene-flow with conspecific wild populations [65]. To allow the understanding of the processes at play requires contextualization of the niche constructing behaviours of human communities [20]. The framework considers potential pathogen flow within emergent farming systems, and key determinants of disease highlighted above and summarized in [66]. It draws from conceptualization of wildlife health as a cumulative effect that changes through time [16], whilst also remaining context dependent. It also allows articulation with conceptualizations of the evolution of agriculture in a similar cumulative way, e.g. cumulative cultural evolution, where certain innovations or ideas are selectively retained and built on through successive human generations [24].

Moving beyond anthropocentrism is a key dimension of One Health principles. A wider and more inclusive consideration of species interactions, outside relationships with humans, may help to frame ecological interactions not typically considered. In this way, improving conceptual frameworks can help provide guidance to research strategy that coherently links to the overarching research goals [14]. Research in recent years has demonstrated examples where some animal species implicated in ancient disease transmission have gone unnoticed until identified through aDNA approaches, such as the introduction of tuberculosis to the Americas by seals [67] and the role of squirrels as a host of leprosy in mediaeval England [41]. Clarified and inclusive frameworks that formulate past disease ecology within broader multispecies, socio-ecological models thus have a role in framing research topics and sample selection (i.e. selecting species/populations in which pathogen occurrence should be investigated) (Fig. 4). For example, modelling of zoonotic brucellosis indicated it would have become endemic in early managed livestock in the Early Neolithic of Southwest Asia [68], which has subsequently been ground-truthed through palaeogenomic analysis [34]. Even small domestic populations, such as carnivores (i.e. dogs and cats), can have a significant impact on local fauna, with the potential to modify or disrupt ecosystems well beyond human-occupied spaces [69, 70], including enabling pathogen transmission across multiple species (Fig. 4). As an archaeological example, Susat *et al*. [71] have demonstrated the potential of dogs to be carriers of *Yersinia pestis* and likely establishing epidemiological connections between local micromammal reservoirs and human communities in Late Neolithic Germany.

Contemporary wildlife farming contexts are not direct analogues for early domestication scenarios. They are employed here to help explore the factors at play and to set up productive contexts for thinking about the distributed health impacts of farming decisions and ecological interactions. Animals are often raised in intensive contexts; different from the low yield, low intensity emergent systems theorized in archaeological context. Also, health and welfare information on these systems is relatively scarce, although it is known that predisposition to disease emergence in such populations can be shaped by intensive farming conditions causing stress and immunosuppression [23]. In addition, a high host density promotes transmission, and high turnover in the supply of susceptible hosts [72]. The aim here is to ensure that how we think of past systems can be applied to contemporary scenarios [24]. Consideration of contemporary wildlife farming scenarios may help us consider a wider constellation of socio-ecological and epidemiological relationships to frame the pathways to agriculture and its impacts. Domestication as a process is often conceptualized as a linear evolutionary model, reducing the range of possible relationships open to examination, which in practice may have been more experimental and random [73]. Viewing domestication in a broader lens may more effectively encompass categories of ‘low-level food production’ [7].

A long-term comparative perspective may also bring focus to modern contexts. Contemporarily, Western societies, in particular, tend to view wild and domestic animals in short-term, static perspectives as clearly delineated immutable categories [40]. Somewhat offset from this, and often receiving less societal scrutiny and valorization is the captive breeding of wild animals [22]. We argue that greater understanding of these populations may be rendered from viewing them in a long-term and comparative One Health framework. This is important for their welfare, and that of human communities, given the ongoing risks of zoonotic transfers [74].

Understanding long-term multispecies interactions and gene-culture coevolutionary processes are important for public health. This enables understanding of how human decisions and cultural actions can have evolutionary effects. For example, in the case of Marek’s disease virus (MDV) in chickens, palaeogenomic research indicates that it has been circulating for at least 1000 years, but that ancient MDV strains were substantially less virulent than modern ones, with a change in virulence linked to modern intensive management conditions and imperfect (‘leaky’) vaccines [75]. As noted above, Early Neolithic livestock management decisions promoted conditions for endemicity of brucellosis in farmed animals [68] and a permanent risk to human populations. In a multispecies farming context, this process would also lead to the speciation and host adaptation of the pathogen [34]. A long view perspective is important for understanding the contexts of current health risks, especially when those contextual factors emerged and accumulate at different times in history. The emergence of avian influenza H5N1 and H7N9 in China, for example, has been linked to intensifications of rice farming, the integrated duck-rice farming system and intensive chicken production. Understanding their cumulative effects is essential for designing structural interventions [72]. Biocultural coevolutionary processes were an important factor shaping infectious disease risk 10 000 years ago; they are also important today, as visible in the evolution of antimicrobial resistance in response to human antimicrobial treatment strategies and usage [30, 76].

## CONCLUSIONS

We all live in a multispecies world. The nature and frequency of connections between these species shapes the ecology and evolution of infectious diseases. Restructuring human-animal relationships through time has provided opportunities for spillover and maintenance of parasites in different directions and different species populations. Through the Holocene, the strongly niche constructing behaviours of humans have significantly shaped these processes, as seen clearly in the emergence and intensification of domestic animal husbandry. Public and animal health scientific communities are still battling the infectious diseases that emerged with the transition to farming, and humans are still re-shaping connections and ecologies creating conditions for the emergence of new pathogens.

This paper has sought to bring together the study of modern and ancient human-animal relationships, linking knowledge on the processes of prehistoric animal domestication with that on contemporary wildlife farming. It contributes a framework for the long view understanding of intensifying wild animal management, which demonstrates the contribution of the humanities, and archaeology in particular, to improving understanding and contextualization of a contemporary One Health issue. It explores modern comparative case studies for helping us think about early farming and domestication processes. Improved understanding of wildlife farming has potentially significant implications within ethical, legal and public health spheres. De-centering humans in the story, through One Health approaches, will help us move focus from pair-wise relationships to community ecology and well-being.

Through situating contemporary wildlife farming in a wider conceptual framework, the paper allows understanding of key socio-ecological features that can increase disease risk and contribute to evolutionary dynamics of human and animal pathogens. Recognition of these features can inform the design and targeting of effective disease mitigation strategies. For example, enhancements in the management and regulation of wildlife farming should include the reduction of high animal densities, which facilitate pathogen transmission, and an emphasis on improving animal health and immunity through better welfare and husbandry practices. Fuller understanding and appreciation of all interspecies interfaces and the role human cultural practices play in such ecological and evolutionary contexts is also important for developing effective prevention strategies for diseases associated with wildlife farming.

## Data Availability

No new data were generated for this study.
